# CicerSpTEdb: A web-based database for high-resolution genome-wide identification of transposable elements in *Cicer* species

**DOI:** 10.1371/journal.pone.0259540

**Published:** 2021-11-11

**Authors:** Morad M. Mokhtar, Alsamman M. Alsamman, Haytham M. Abd-Elhalim, Achraf El Allali

**Affiliations:** 1 African Genome Center, Mohammed VI Polytechnic University, Ben Guerir, Morocco; 2 Agricultural Genetic Engineering Research Institute, Agricultural Research Center, Giza, Egypt; Ben-Gurion University, ISRAEL

## Abstract

Recently, *Cicer* species have experienced increased research interest due to their economic importance, especially in genetics, genomics, and crop improvement. The *Cicer arietinum*, *Cicer reticulatum*, and *Cicer echinospermum* genomes have been sequenced and provide valuable resources for trait improvement. Since the publication of the chickpea draft genome, progress has been made in genome assembly, functional annotation, and identification of polymorphic markers. However, work is still needed to identify transposable elements (TEs) and make them available for researchers. In this paper, we present CicerSpTEdb, a comprehensive TE database for *Cicer* species that aims to improve our understanding of the organization and structural variations of the chickpea genome. Using structure and homology-based methods, 3942 *C*. *echinospermum*, 3579 *C*. *reticulatum*, and 2240 *C*. *arietinum* TEs were identified. Comparisons between *Cicer* species indicate that *C*. *echinospermum* has the highest number of LTR-RT and hAT TEs. *C*. *reticulatum* has more Mutator, PIF Harbinger, Tc1 Mariner, and CACTA TEs, while *C*. *arietinum* has the highest number of Helitron. CicerSpTEdb enables users to search and visualize TEs by location and download their results. The database will provide a powerful resource that can assist in developing TE target markers for molecular breeding and answer related biological questions.

**Database URL:** http://cicersptedb.easyomics.org/index.php

## Introduction

Transposable elements (TEs) are mobile DNA sequences that can move and integrate themselves in another location throughout the genome [1]. Based on the transposition systems, TEs were classified into two classes [2]. Class I is known as retrotransposons, and Class II is known as DNA transposons. Retrotransposons utilize a copy and paste system, while DNA transposons use the cut and paste systems to transpose along the genome [2]. Retrotransposons are divided into two sub-classes, the long terminal repeat-retrotransposons (LTR-RT) and the non-LTR retrotransposons [2]. More evidence documented that TEs contribute to the reshaping of plant genomes and play important roles in regulating, altering, and creating new genes, as well as its essential role in the evolutionary dynamics of host genomes [3–5]. Many reports discussed in detail the impacts of TEs in both the genome and the epigenome [6, 7], the creation of pseudo-gene [8], the alteration [8], and transcriptional silencing [9, 10]. Moreover, TEs affect the development of both vertebrates [11] and plants [12]. In rice, maize, wheat, and barley, there is a correlation between the insertion of TEs near genes and the increased mutation rates in regulatory regions and coding sequences [13]. TEs represent a large percentage of plant genomes, such as rice 40% [14], maize, and wheat 85% [15, 16]. In plants, researchers have found evidence that TEs affect agronomic traits for maize [17], grape [18], foxtail millet [19], blood oranges [20], apples [21, 22] and others. Since TEs play an important role in genome variations, their genetic variation could be considered advantageous for crop breeding [23–26].

The *Cicer* genus contains 45 species with nine annual and 36 perennial species. Only chickpea (*Cicer arietinum* L.) is cultivated in 49 countries on a large scale. Currently, only two annual *Cicer* species (*Cicer reticulatum* and *Cicer echinospermum*) are in the primary and secondary gene pools and crossable to chickpea [27]. Chickpea is one of the most important Fabaceae crops. It has special significance to food security in developing countries due to its potential nutritional and health benefits [28]. According to 2019 FAO statistics [29], about 13.7 million hectares were cultivated with chickpea in more than 47 countries, yielding about 14.2 million tons. As a member of the Fabaceae family, chickpeas can restore soil fertility by fixing atmospheric nitrogen [30]. Because of its value for the economy and human nutrition, chickpea-related research has also increased interest, especially in crop improvement, genetic, genomics, and basic biological studies [31].

A significant achievement in *Cicer* species genomics was attained as a result of publishing the genomic sequences of *Cicer arietinum* [30–32], *Cicer reticulatum* [33], and *Cicer echinospermum* [34]. Chickpea genomic studies aim to improve our knowledge of genome organization, structural variations, genome evolution, and the basic biology of legume crops. Advances in bioinformatics and sequencing technologies have led to the fast creation of large-scale sequencing and genotyping data sets for chickpea [35–37]. The integrated study of massive phenotypic and genomics data opens the door for discovering new genes, functional elements, and biological processes correlated with several economic traits [38].

The fast-growing of chickpea omics data led to the establishment of many functional genomics databases, including the microsatellites markers database "CicArMiSatDB" [39], the SNP and InDels database "CicArVarDB" [40], and the transcriptome database "CTDB" [41]. In recent years, functional genomic elements such as miRNAs [42], transcription factors [38], long non-coding RNAs [43], and transposable elements [44] were discovered for several plant species. For chickpea, miRNAs were identified in 2014 [45], transcription factor in 2016 [46], and long non-coding RNAs in 2017 [47].

At present, several multiple-species TE databases are available and are exemplified by Repbase [48], GyDB [49], PlantsDB [50], and RepetDB [51]. Species-specific TE databases are also available such as RetrOryza [52], BmTEdb [53], BrassicaTED [54], MnTEdb [55], FmTEMDb [56], PlanTE-MIR DB [57], SPTEdb [58], and ConTEdb [59]. Despite advancements in functional genome annotation of chickpea, no database for chickpea TEs has been established. Chickpea TEs need to be clearly identified in detail and made available to researchers. Studying these valuable genomic elements should accelerate the improvement of this important crop and become a new area of research in chickpea.

Genome-wide identification of TEs in the *Cicer* species and the establishment of comprehensive TE databases are key resources for the accurate characterization of genes and other genomic elements. Here, we used the Extensive *de-novo* TE Annotator (EDTA) pipeline [60] as both structure and homology-based methods to identify, classify and annotate TEs in *Cicer* species. All identified TEs were deposited for browsing and visualization in the developed *Cicer* species Transposable Elements database (CicerSpTEdb). CicerSpTEdb will represent an open resource that will allow researchers to improve our knowledge of the origin, organization, structural variations, and evolution of the *Cicer* species, including the chickpea genome. The CicerSpTEdb database will also provide an essential resource to other related legume crops. In addition, we hope that CicerSpTEdb aid plant breeders in developing TE target markers for molecular breeding and help the research community in general answer related biological questions.

## Materials and methods

### Genomic data

We retrieved the chickpea (*C*. *arietinum*) reference genome sequence of Kabuli type cultivar CDC-Frontier (ASM33114v1) from the NCBI FTP server [61] in both fasta and gff formats. Due to the unavailability of annotations, only fasta files were downloaded for *Cicer reticulatum* (GCA_002896235.1) and Cicer *echinospermum* (GCA_002896215.2) from NCBI FTP server [61].

### Identification of TEs

We conducted the intact transposon identification and characterization for the 530, 657, and 715 Mbps representing the *Cicer arietinum*, *Cicer echinospermum*, *and Cicer reticulatum* reference genome, respectively, using EDTA pipeline [60]. EDTA pipeline combines tools for the structure, homology-based, and *de novo* identification methods. The EDTA pipeline combines LTRharvest [62], LTR_FINDER [63], LTR_retriever [64], Generic Repeat Finder [65], TIR-Learner [66], HelitronScanner [67], and RepeatModeler [68]. The parameters of each tool are described in S1 File.

### Estimation of LTR-RT insertion time

ClustalW [69] was used to alignment the 5′and 3′ LTRs of each intact LTR-RTs to estimate the insertion time of LTR-RTs. The nucleotide substitutions/divergence among LTRs (K) were computed by applying the Kimura-2-parameter model [70] using the KaKs_Calculator program [71]. Using an evolutionary rate (r) of 1.5 × 10^−8^ substitutions per synonymous site per year [72–74] and the formula T = k/2r the insertion time was estimated [70].

### Identification of TEs positioned inside or nearby genes

Perl scripts were used to differentiate the predicted TEs according to the localization in the genome sequence. The goal was to identify TEs that are positioned within or nearby genes according to the genome annotation. For nearby genes, 10 kbp upstream genes were used to detect TEs located inside this region. We performed gene ontology on all genes that house TEs using UniProtKB [75].

### Protein-protein interaction analysis

The amino acid sequences of genes that contain or are close to TEs were used for protein-protein interaction analysis using the STRING database [76]. Cytoscape 3.8.2 software and the STRING-App were used for PPI networks analysis and visualization [77, 78].

### Database construction

**Fig 1:**
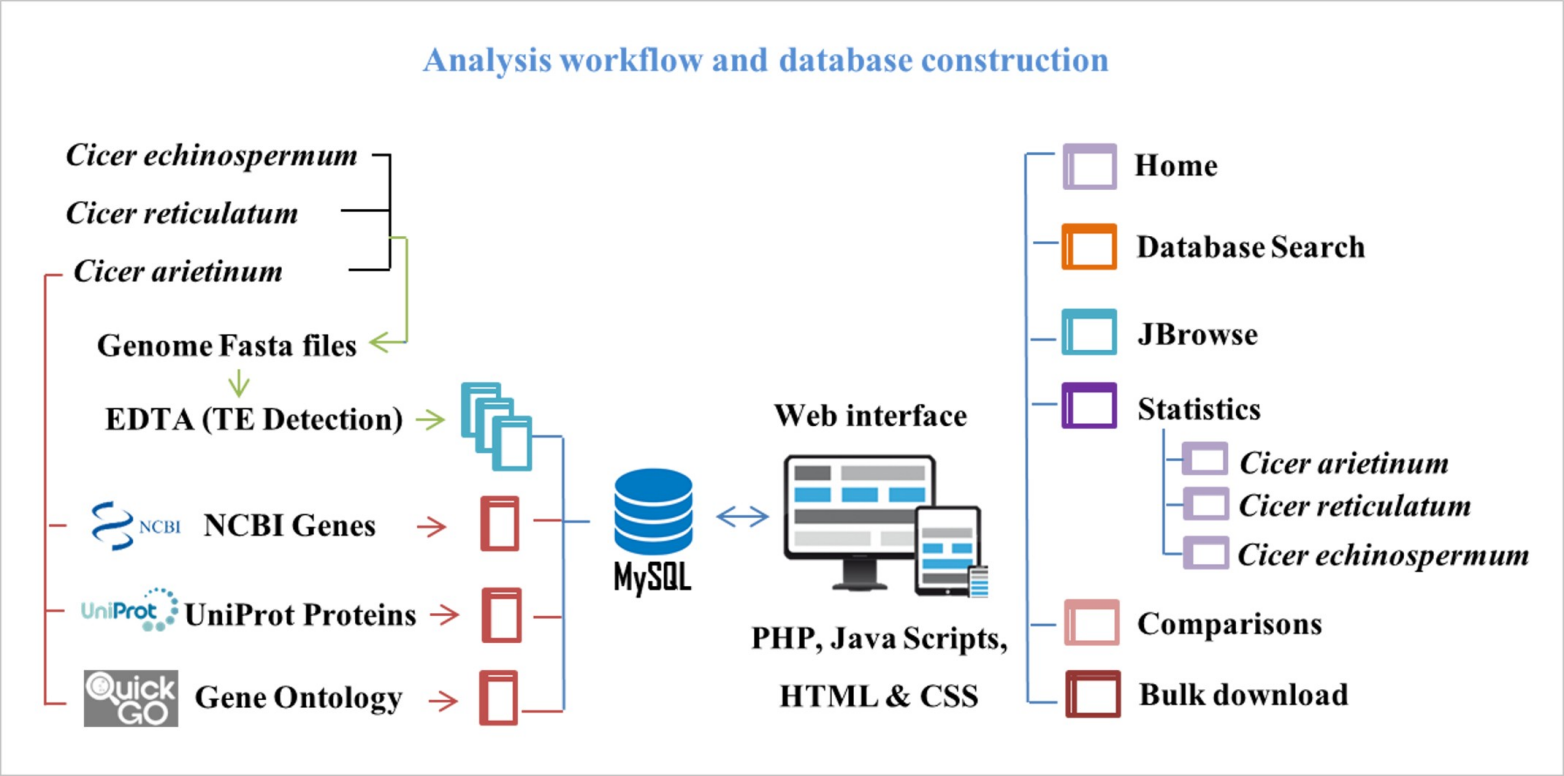
The overall framework used for identifying and characterizing TEs in *Cicer* species and the steps involved in creating CicerSpTEdb.

JBrowse [79] was embedded in our developed database to map and visualize identified TEs across the reference genome. The CicerSpTEdb database was designed as an interactive web application using CSS, Perl, MySQL, PHP, HTML, and JavaScript. (Fig 1) illustrates the framework used to identify TEs in *Cicer* species and develop the proposed CicerSpTEdb.

## Results and discussion

TEs identification and annotation have been formed for numerous plant genomes through extensive efforts and manual identification, e.g., Arabidopsis [80], rice [14], and maize [81]. Despite the increase in the number of sequenced plant genomes, manual identification remains labor-intensive, and automated TE annotation is needed [60]. Intact TEs are the complete structures of TEs that can transpose throughout the genome [82]. Most sequenced plant genomes have had annotated TEs. However, it is important to predict which of these TEs are still viable for mobility. Thus, in the present investigation, we focused only on the analysis of intact TEs. Using the EDTA pipeline [60], several approaches were applied to identify intact TEs in the *C*. *arietinum*, *C*. *reticulatum*, and *C*. *echinospermum* genomes. EDTA consist of LTRharvest [62] and LTR_FINDER [63] for LTR identification. False discoveries are filtered by LTR_retriever [64]. In addition, TIR-Learner [66] is used for TIR candidates identification, and HelitronScanner [67] is used to recognize Helitron candidates.

### TEs identification in *Cicer* species

As a result, a total of 794 intact LTR-RTs were identified in *C*. *arietinum*, including 521 Copia, 80 Gypsy, and 193 unknown LTRs. For DNA TEs, we identified 775 Mutator followed by 245 CACTA, 215 hAT, 156 helitrons, 28 Tc1 Mariner, and 27 PIF Harbinger (Table 1). S1 Table includes details of the identified TEs, *including the* chromosome/scaffold id, TE start and end position in the genome, TE corresponding superfamily, and TE length.

**Table 1:** Summary of the intact TEs identified in *Cicer* species.

TE superfamily	*Cicer arietinum*			*Cicer reticulatum*			*Cicer echinospermum*		
	No. elements	Total length (bp)	Percentage of the genome (%)	No. elements	Total length (bp)	Percentage of the genome (%)	No. elements	Total length (bp)	Percentage of the genome (%)
Copia	521	2692203	0.507	1097	7294492	1.02	1404	10055403	1.53
Gypsy	80	466654	0.088	267	1983455	0.277	423	3515110	0.535
Unknown LTR	193	578105	0.109	407	1514753	0.212	445	1766078	0.269
CACTA	245	594115	0.112	329	822381	0.115	291	757423	0.115
hAT	215	211394	0.04	211	231880	0.032	263	274877	0.042
Helitron	156	1498699	0.282	151	1142623	0.16	152	1438982	0.219
Mutator	775	1001091	0.189	1025	1397305	0.195	892	864817	0.132
PIF Harbinger	27	21975	0.004	56	100906	0.014	40	52275	0.008
Tc1 Mariner	28	40588	0.008	36	56626	0.008	32	43869	0.007
Total	2240	7104824	1.339	3579	14544421	2.033	3942	18768834	2.857

**Fig 2:**
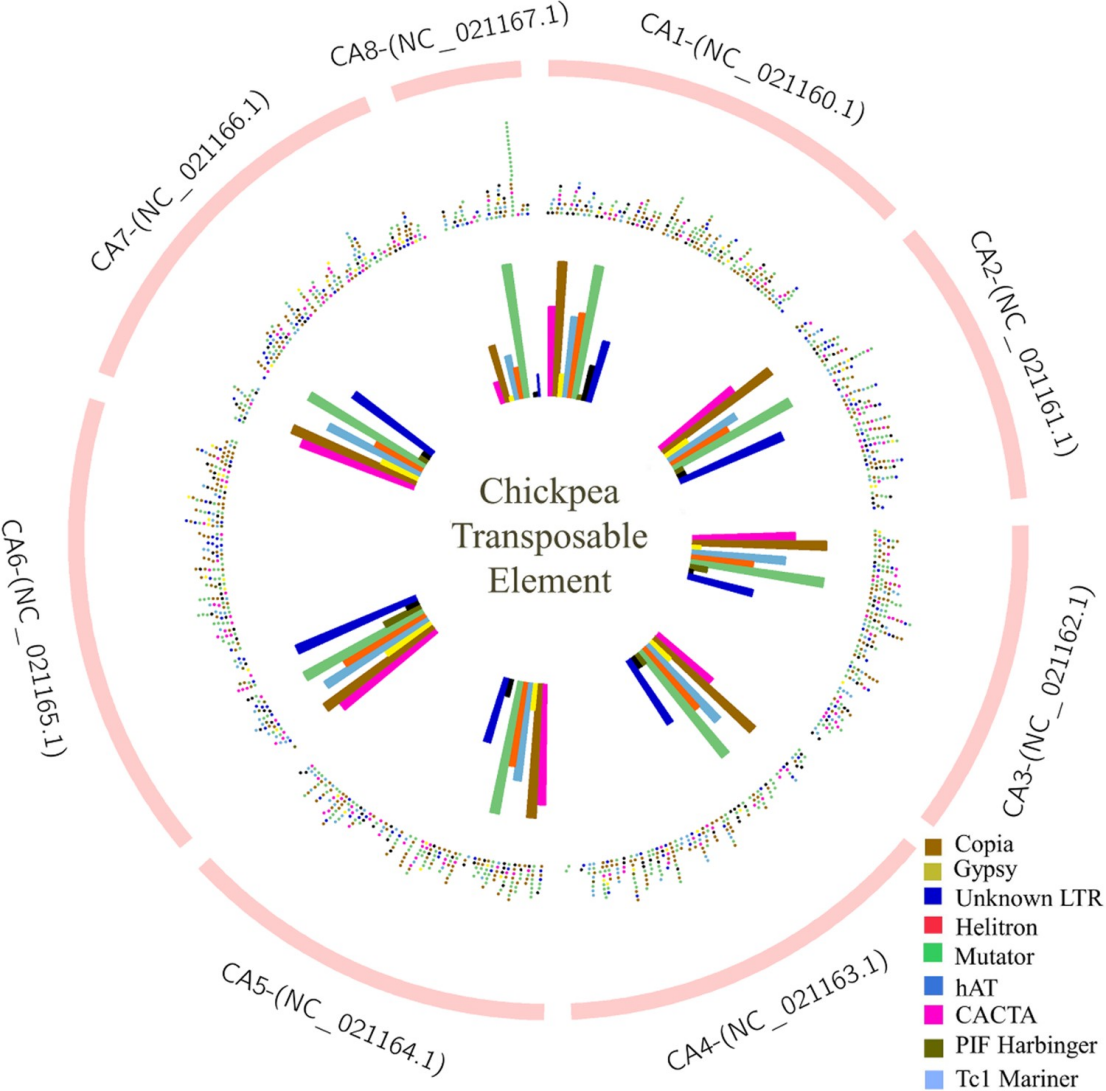
The distribution of intact TEs across eight chickpea chromosomes. The outermost circle in pink-colored represents eight *C*. *arietinum* chromosomes (CA1 to CA8). The middle circle illustrates the distribution of TE types in different colors, and the innermost circle shows the histogram of TE types in each chromosome.

Interestingly, Varsheny et al. [30] reported that approximately 49.41% of the *C*. *arietinum* genome is composed of TEs and unclassified repeats, including 617,505 repeat retrotransposons and 197,959 DNA transposons. However, our investigation produced lower numbers and found that the intact TEs represent only 2240 elements, approximately 1.3% of the whole genome. This difference could be because many of them will not be intact TEs and may be nested elements or fragmented.

(Fig 2) shows the distribution and histogram of TEs across eight *C*. *arietinum* chromosomes. As shown, the distribution of TEs superfamily were 217, 179, 187, 200, 230, 240, 200, 76 elements for chromosomes 1, 2, 3, 4, 5, 6, 7, and 8, respectively. Chromosome 6 (CA6) had the highest presence of TEs (240 elements), including 57 Copia, 55 Mutator, 29 unknown, 27 CACTA, 27 hAT, 21 helitrons, 12 Gypsy, 9 PIF, and 3 Tc1_mariner.

The analysis of *C*. *reticulatum* TEs revealed that 14.54 Mb (approximately 2% of the genome) were intact TEs. The highest copy number of LTR-RT was 1097 Copia, followed by 193 unknown LTRs, and 80 Gypsy. In addition, 1808 *C*. *reticulatum* DNA transposons were detected and consist of 1025 Mutator, 329 CACTA, 211 hAT, 151 helitrons, 56 PIF Harbinger, and 36 Tc1 Mariner (Table 1 and S2 Table). For *C*. *echinospermum*, a total of 3942 intact TEs were detected and covered 18.76 Mb (approximately 2.8% of the genome). Out of these, 2272 LTR-RTs included 1404 Copia, 423 Gypsy, and 445 unknown LTRs. *C*. *reticulatum* has more Mutator, PIF Harbinger, Tc1 Mariner, and CACTA TE types (Table 1 and S3 Table). The present investigation revealed that *C*. *reticulatum*, *C*. *echinospermum*, and *C*. *arietinum* have a higher copy number of Copia superfamily than Gypsy, which is consistent with previous reports of flax [73], grape [83], cocoa [84], and cucumber [85].

To our knowledge, there are no TE reports for both *C*. *echinospermum and C*. *reticulatum* to allow the discussion of our new findings. In addition, the draft genomes of *C*. *reticulatum*, *C*. *echinospermum*, and *C*. *arietinum* were partially sequenced. The sizes of their available sequences are 530, 657, and 715 Mb, respectively. This variation in size could be correlated with the variation of the identified copy number of TEs in these genomes.

### Estimation of LTR-RT insertion time

It is deemed that the 5′ and 3’ LTRs are the same at transposition time for each LTR-RT. Consequently, based on nucleotide substitutions/divergence among LTR-RT, the 5’ and 3’ LTRs accumulated through ages were applied to estimate the insertion time [86–88]. In the present investigation, the 5′ and 3’ LTR nucleotide substitutions were used to estimate the identified intact LTR-RT insertion time across *Cicer* species.

**Fig 3:**
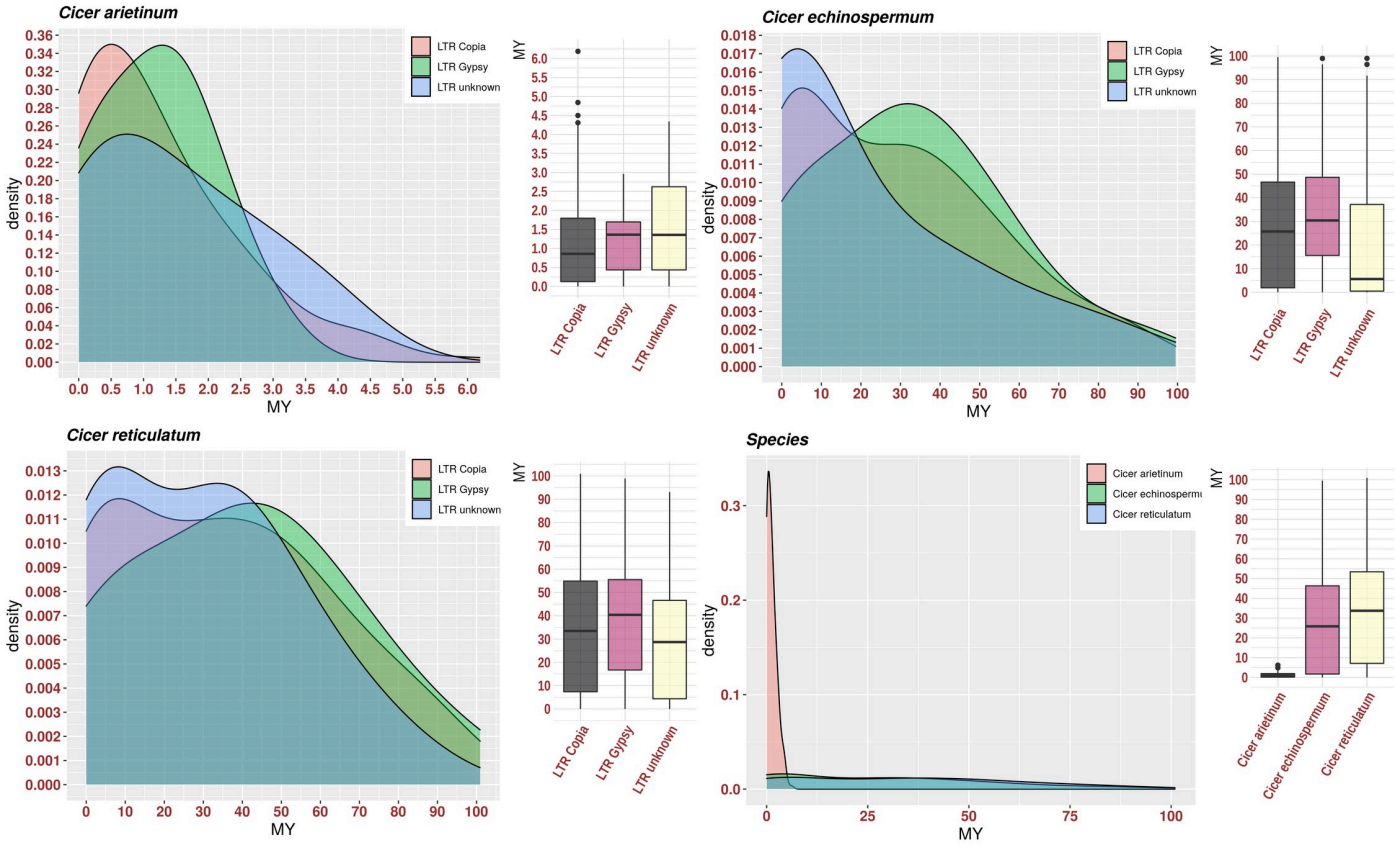
Estimation of *Cicer* species LTR-RT insertion age in millions of years (My).

For *C*. *arietinum*, the minimum and maximum assumed age after discarding outliers using boxplot analysis ranged from 0 to 4.4 million years (MY) with an average of 0.94 MY. The unknown elements were older than Copia and Gypsy (4.3, 4.2, and 2.9 MY, respectively). The average insertion ages of the unknown, Copia and Gypsy elements are 1.3, 1.3, and 0.85 MY, respectively (Fig 3). Interestingly, about 23.3% of Copia, 21.7% of Gypsy, and 23.4% of unknown elements have estimated ages of < 0 MY, and they may still be active elements. While the proportions of insertion times that are more than 1.2 MY were 56.5%, 53.1%, and 40% of Gypsy, unknown, and Copia, respectively (Fig 3).

For *C*. *reticulatum*, the estimated age ranged from 0 to 100 MY with an average of 33.7 MY. The unknown elements are younger than Copia and Gypsy elements. The Copia, Gypsy, and unknown elements’ average insertion times were 33.4, 40, and 28.7 MY, respectively. Overall, about 10.5% of Copia, 8.9% of Gypsy, and 7.5% of unknown elements were estimated to have an age of < 0 MY. While the proportions of insertion times that are more than 1.2 MY were 86%, 85.6%, and 81.9% of Gypsy, Copia, and unknown elements, respectively (Fig 3).

For *C*. *echinospermum*, the estimated age ranged from 0 to 99.5 MY with an average of 25.8 MY. The unknown elements are younger than Copia and Gypsy elements. The average insertion time of the Copia, Gypsy, and unknown elements were 30.3, 5.5, and 25.7 MY, respectively. Overall, about 6.6% of Gypsy, 12.5% of Copia, and 21.7% of unknown elements had an estimated age of < 0 MY. While the proportions of insertion times that are more than 1.2 MY were 87.5%, 78.7%, and 62.6% of Gypsy, Copia, and unknown elements, respectively (Fig 3).

The chromosomes number of the *C*. *arietinum*, *C*. *echinospermum*, and *C*. *reticulatum* were the same 2n = 16. Therefore *C*. *reticulatum* was in the primary gene pools and recognized as the wild ancestor of the *C*. *arietinum*. In addition, genetic studies revealed that the *C*. *echinospermum* was closely and in secondary gene pools of *C*. *arietinum* [27, 89]. The estimation of *Cicer* species LTR-RT insertion time revealed that the wild species *C*. *echinospermum* and *C*. *reticulatum* were older than the cultivated species *C*. *arietinum* (Fig 3). Based on estimated LTR-RT age, *C*. *arietinum* may be derived/split from their wild progenitor *C*. *reticulatum* ~ 4.4–6 MY.

### TEs length distribution

**Fig 4:**
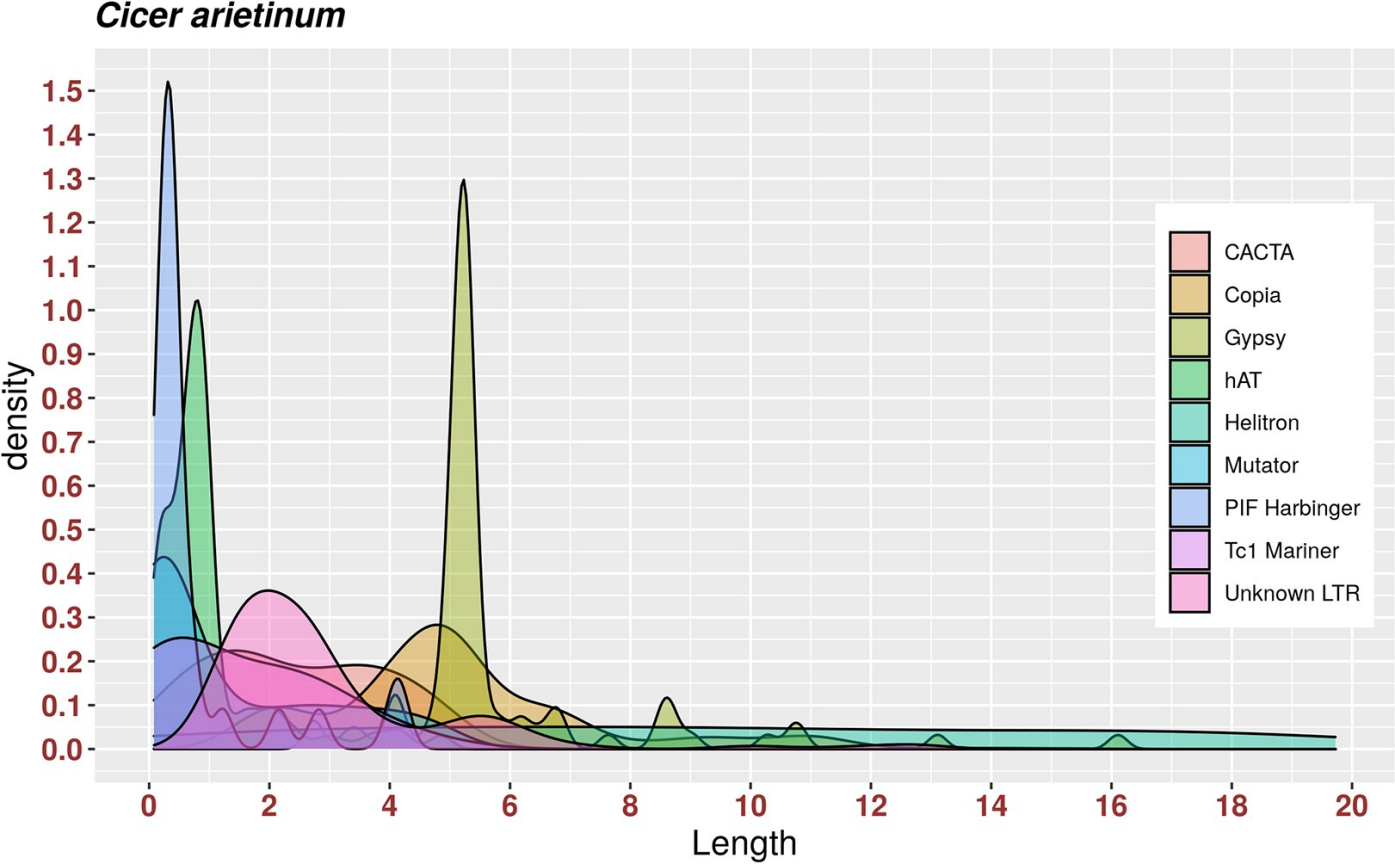
The distribution of *C*. *arietinum* TEs superfamilies according to their length.

The lengths of *C*. *arietinum* intact TEs ranged from 80 bp to 19.7 kb for both DNA and LTR transposons. The average sizes of various superfamilies were Gypsy 5.8 kb, Copia 5.1 kb, unknown LTR 2.9 kb, Helitron 9.6 kb, CACTA 2.4 kb, Tc1 Mariner 1.4 kb, Mutator 1.2 kb, hAT 0.9 kb, and PIF Harbinger 0.8 kb (Fig 4). For *C*. *echinospermum* TEs, lengths ranged from 80 bp to 19.6 kb for DNA and LTR transposons. The average sizes of various superfamilies were Gypsy 8.3 kb, Copia 7.1 kb, unknown LTR 3.9 kb, Helitron 9.4 kb, CACTA 2.6 kb, Tc1 Mariner 1.3 kb, PIF Harbinger 1.3 kb, hAT 1 kb, and Mutator 0.9 kb (S1 Fig). For *C*. *reticulatum*, TE lengths ranged from 80 bp to 21.7 kb for both DNA and LTR transposons. The average sizes of various superfamilies were Gypsy 7.4 kb, Copia 6.6 kb, unknown LTR 3.7 kb, Helitron 7.5 kb, CACTA 2.4 kb, PIF Harbinger 1.8 kb, Tc1 Mariner 1.5 kb, Mutator 1.3 kb, and hAT 1 kb (S2 Fig).

### Identification of TEs positioned inside or nearby genes

The transposition of TEs across the genome may affect both nearby genes’ expression and genes unlinked to the insertion. TEs can affect genes through the movement, duplication, and recombination processes, creating new genes or altering the gene structure [86]. Furthermore, they may alter the expression of nearby genes by inserting themself within *cis*-regulatory elements or by presenting a new *cis*-regulatory element that may act as gene enhancers or repressors [90]. Due to the unavailability of annotation for *C*. *reticulatum* and *C*. *echinospermum*, only TEs identified in *C*. *arietinum* were subject to further analysis to determine TEs that are inside or nearby genes.

Overall, 1162 *C*. *arietinum* intact TEs (about 51.8%) were positioned inside (TE-gene chimeras) or nearby genes. Only 20 TEs were found within pseudo-genes (Table 2). From these elements, 426 (approximately 36.6%) were LTR-RT, and 736 (approximately 63.3%) were DNA transposons. For LTR-RT, the Copia superfamily was overrepresented, followed by unknown elements and gypsy superfamilies with 250, 140, and 36 elements, respectively. However, DNA transposons included 326 Mutator, 173 hAT, 150 CACTA, 38 Helitron, 28 Tc1 Mariner, and 21 PIF Harbinger elements.

**Table 2:** Summary of the *C*. *arietinum* TEs that are positioned inside or nearby genes.

TEs superfamily	Number of elements				
	Inside genes (TE-gene chimeras)	Inside pseudo-genes	Near genes (0-2kb)	Near genes (2-10kb)	Other genomic regions
Copia	100	5	29	116	271
Gypsy	27	1	1	7	44
Unknown LTR	91	2	3	44	53
CACTA	54	5	14	77	95
hAT	70	3	28	72	42
Helitron	9		10	19	118
Mutator	92	4	53	177	449
PIF Harbinger	9		1	11	6
Tc1 Mariner	19		1	8	
Total	471	20	140	531	1078

More evidence documented that TEs construct the chimeric genes (TE-gene chimeras) in plants [8, 91]. Previous eukaryotic reports revealed that one thousand human proteins contain TEs [92, 93], and few expressed genes house TEs in Drosophila [94]. In addition, approximately 1.2% of Arabidopsis proteins were constructed from TE-gene chimeras [95]. Consistent with our results, previous studies reported that Class I TEs favor transposition inside gene-poor heterochromatic regions [96]. In comparison, euchromatin regions have more Class II TEs that prefer to transposition inside or nearby genes [97–99]. The finding that TE elements inside and nearby genes in *C*. *arietinum* are overrepresented by Copia than Gypsy is consistent with previous studies in maize [100], Arabidopsis [95], and sugarcane [101].

Regarding class II TEs, Mutator was overrepresented, followed by hATs, while Helitrons were underrepresented. Our results agree with Lockton et al. [95] and Leonardo et al. [73], who found that hATs were overrepresented in Arabidopsis, while Helitrons were underrepresented in flax. Interestingly, the Mutator superfamily was overrepresented inside and closely to genes in *C*. *arietinum*. More evidence documented that the association between Mutator elements and genes supports TE-mediated gene transposition in rice [91] and Arabidopsis [102]. Finally, the distance between identified TEs near genes and genes ranged from 3 to 9.9 kb (S4 Table). From these TEs, 140 elements were located within 2kb near genes, among this 53 Mutator, 29 Copia, 28 hAT, 14 CACTA, ten Helitron, three Unknown_LTR, 1 Gypsy, 1 PIF_Harbinger, and 1 Tc1_Mariner.

### Functional classification by gene ontology analyses

**Fig 5:**
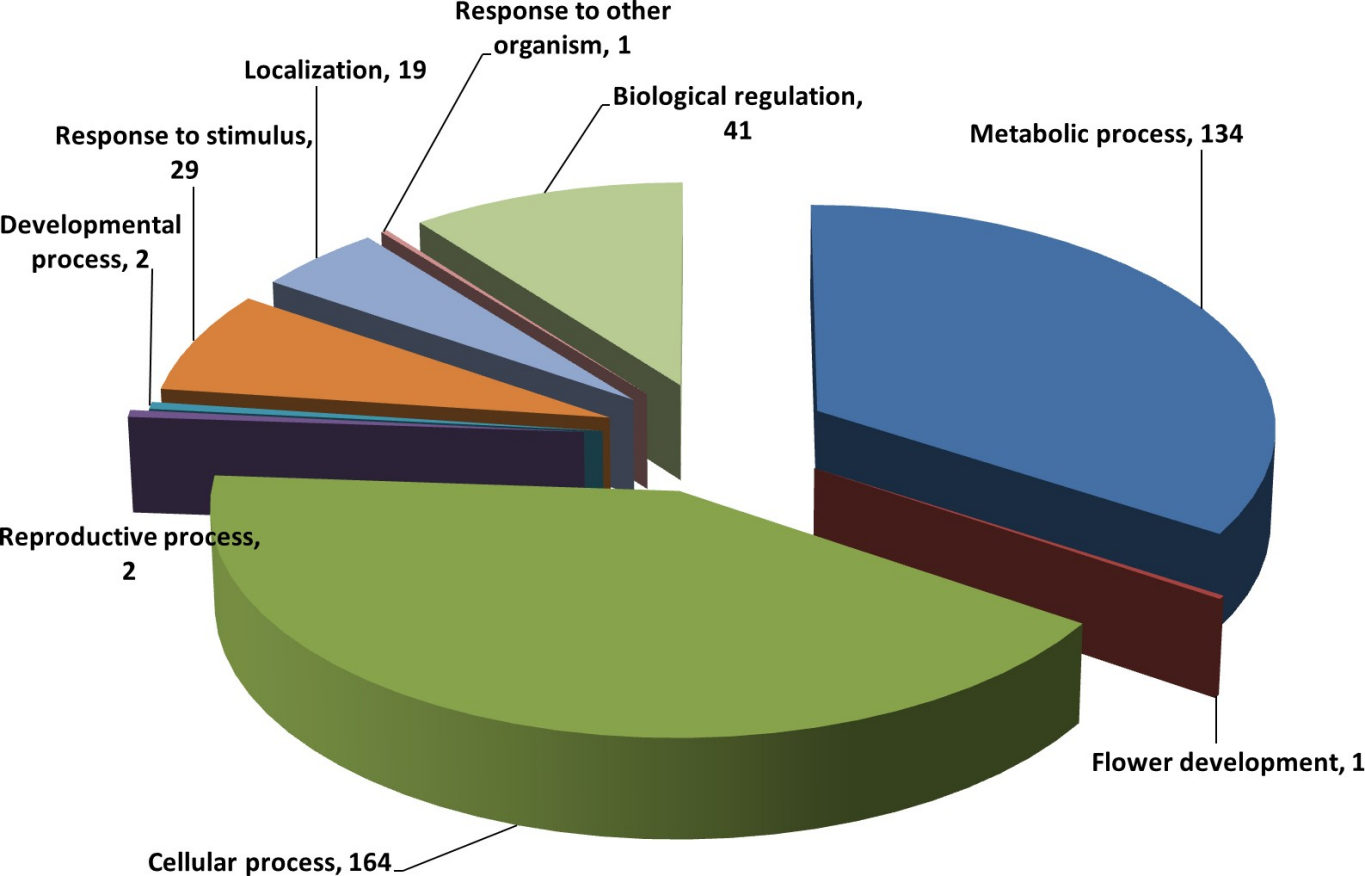
Gene ontology of 213 *C*. *arietinum* TE-gene chimeras assigned to 393 biological processes GO terms.

To determine whether the genes housing TEs in their sequence were disrupted or still have functions, UniProtKB [75] was used to map and classify 441 TE-gene chimeras according to their function (GO terms). Only 366 genes were successfully mapped to 482 UniProtKB IDs and assigned to GO terms. These GO terms include 315 genes assigned to 486 molecular functions GO terms (S3 Fig), 213 genes assigned to 393 biological processes GO terms (Fig 5), and 215 genes assigned to 325 cellular components GO terms (S4 Fig). The 393 GO terms assigned to biological processes were distributed among 164 cellular process, 134 metabolic process, 41 biological regulation, 29 response to the stimulus, 19 localization, two reproductive processes, two developmental processes, one flower development, and one response to another organism (Fig 5). Molecular function analysis showed the overrepresented TE-gene chimeras were catalytic activity, binding, and transporter activity. In contrast, the underrepresented TE-gene chimeras were DNA-binding transcription factor activity and Structural constituent of ribosome (S3 Fig). Based on these results, we can infer that a high percentage of TE-gene chimeras are still functional in various biological processes in *C*. *arietinum*. However, to determine their level of activity, further experimental validation still needs to be performed.

### Protein-protein interaction analysis

**Fig 6:**
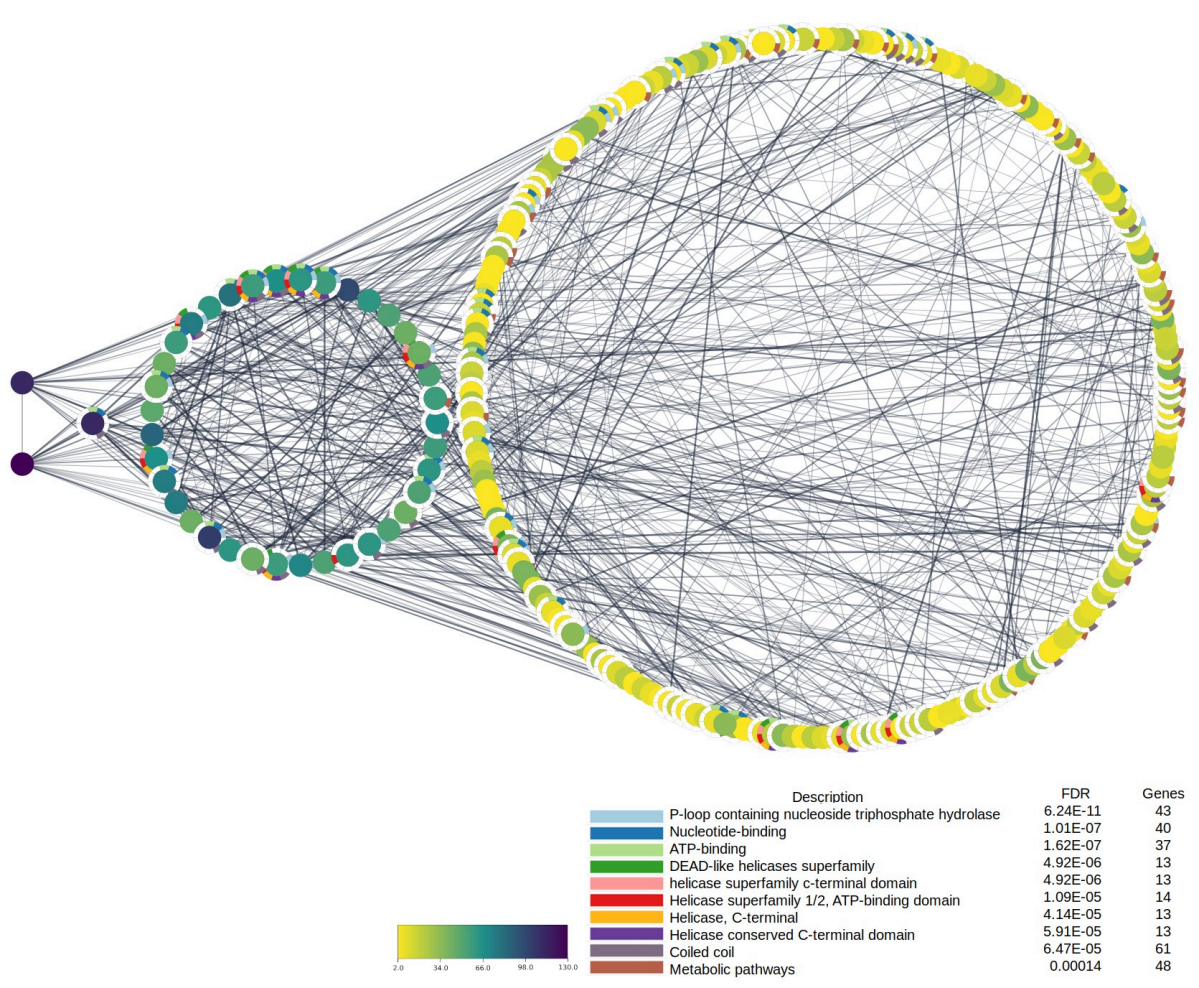
Protein-protein interaction for TE-gene chimeras.

Protein-protein interaction (PPI) analysis is an instrumental analysis tool. It can show how a group of genes interact in the cellular system and their activity level, thus showing their biological importance. Furthermore, it adds more information about the type of connection these proteins have and the biological pathways that they control. We examined the protein interaction activity of chickpea genes that contain or are close to TEs. The STRING database retrieved the interaction information of 619 proteins, from which high interactive proteins could be identified [76]. PPI analysis was carried out for genes that contain, or are close to, TEs, as well as for all genes collectively (Fig 6, S5 and S6 Figs).

The most interactive gene was the DNA-directed RNA polymerase II subunit (RPB1), which contains nearby TEs (Fig 6). The RPB1 gene is an essential component of the RNA polymerase transcription machinery, catalyzing the transcription of DNA into RNA using the four ribonucleoside triphosphates as substrates [103]. Several research articles have discussed the relationship between RPB1 and TEs [104]. The carboxy-terminal domain of eukaryotic RPB1 has a heptad-repeat structure that is intrinsically disordered. These repeats regulate the length of the RPB1 C-terminal domain, which in turn controls transcription activation by influencing transcription cycle coordination. Such a relationship could impact the regulator’s system of essential cellular functions [105]. Phosphoglycerate kinase one gene (PGK1) also revealed many nearby TEs compared to other chickpea genes (S5 Fig). The activity of TEs influences polyploidization modifications in plant genomes, affecting the copy number and the content of genes. Due to its single-copy status per diploid chromosome in several plant species, the PGK1 gene has been widely used to reveal the evolutionary history of complex genomes [106, 107]. The String database offers the ability to analyze PPI networks depending on their biological pathways. It has revealed that the most gene-enriched pathways are those linked to Neclotide binding and metabolic pathways, and mostly, these genes are linked by lab experiments, published articles (text mining), or their genome physical distance (neighborhood) (S6 Fig). Out of these results, we can point out that TEs affect distinctive genes with high interplay activity and consequently impact a widespread biological process in the chickpea genome.

### CicerSpTEdb web interface

The *Cicer* Transposable Elements database (CicerSpTEdb) is accessible through a user-friendly portal (http://cicersptedb.easyomics.org/index.php). The website allows users to explore and understand the Cicer transposable elements. The database offers comprehensive details of TEs and their features in the genome, especially for chickpea. The CicerSpTEdb interface allows users to search, browse, compare, and download TEs interactively. From the homepage, users can capture the essential information about CicerSpTEdb and access relative external databases and software. The navigation bar allows access to six sections for browsing and retrieving data, including Home, Database Search, JBrowse, Statistics, Comparisons, and Bulk Download.

### The Database Search page

**Fig 7:**
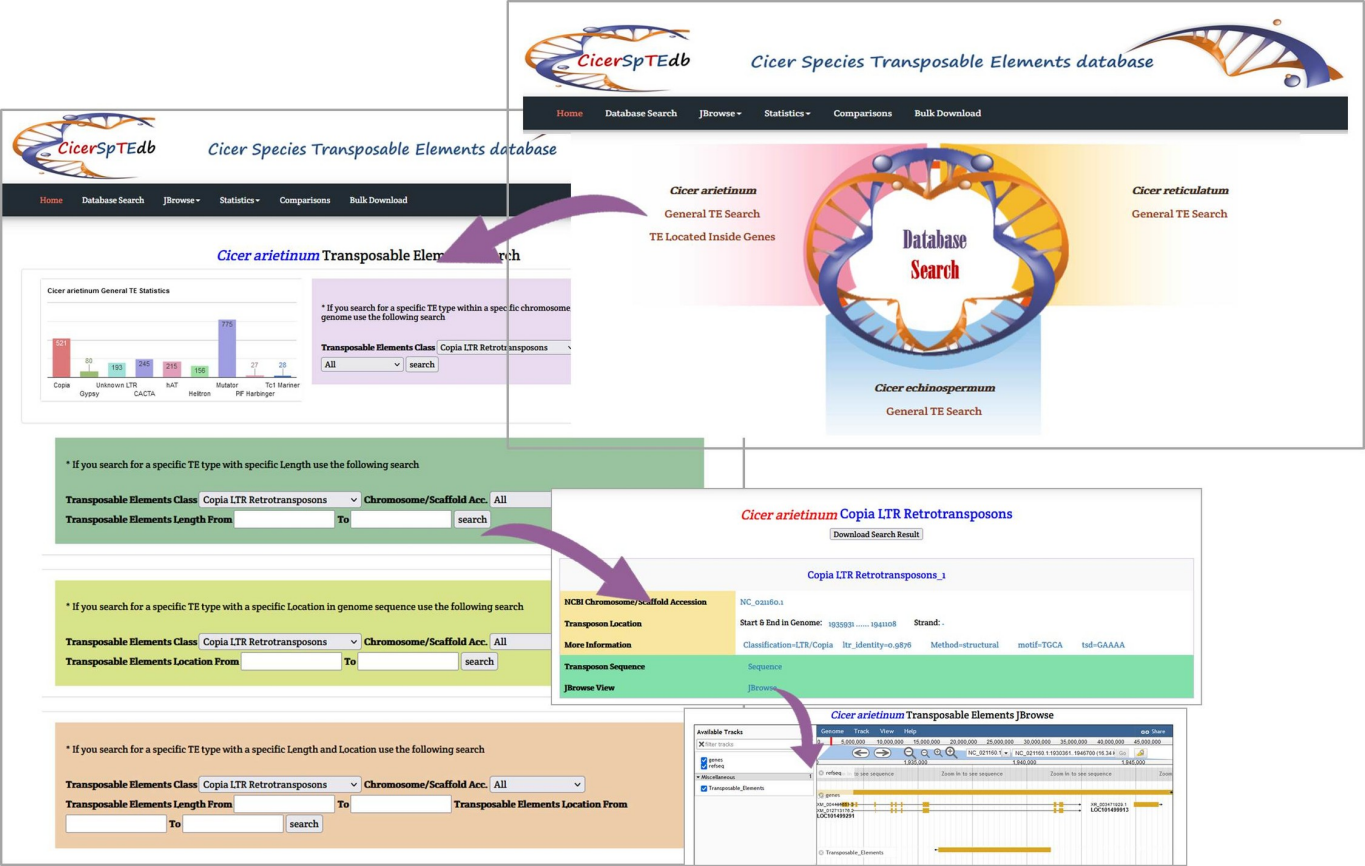
CicerSpTEdb’s TE general search page.

From anywhere on CicerSpTEdb’s interface, users can access the Database Search page through the top bar that links to the main search page. The latter provides links to access two separate *Cicer arietinum* pages. The first page allows a general search of TEs, while the second option provides detailed information on TEs located within genes. In addition, links to access *Cicer reticulatum* and *Cicer echinospermum* TE general search pages are also available. The top section of the TE general search page allows users to see a statistical chart of all identified TEs by type. The main section is divided into four sub-sections. It allows users to 1) search by TE type within a specific chromosome/scaffold or within the whole-genome, 2) search by TE type in a specific chromosome/scaffold or whole-genome with specific TE length, 3) search by TE type with a specific location inside the genome, 4) search by TE type with a specific length and location in the genome. The search results appear on a new page and include NCBI chromosome/scaffold accession, transposon start, end, length, and corresponding strand in the genome, TE structure details, download TE sequence, and a JBrowse link. The results can be exported by clicking the download button (Fig 7).

**Fig 8:**
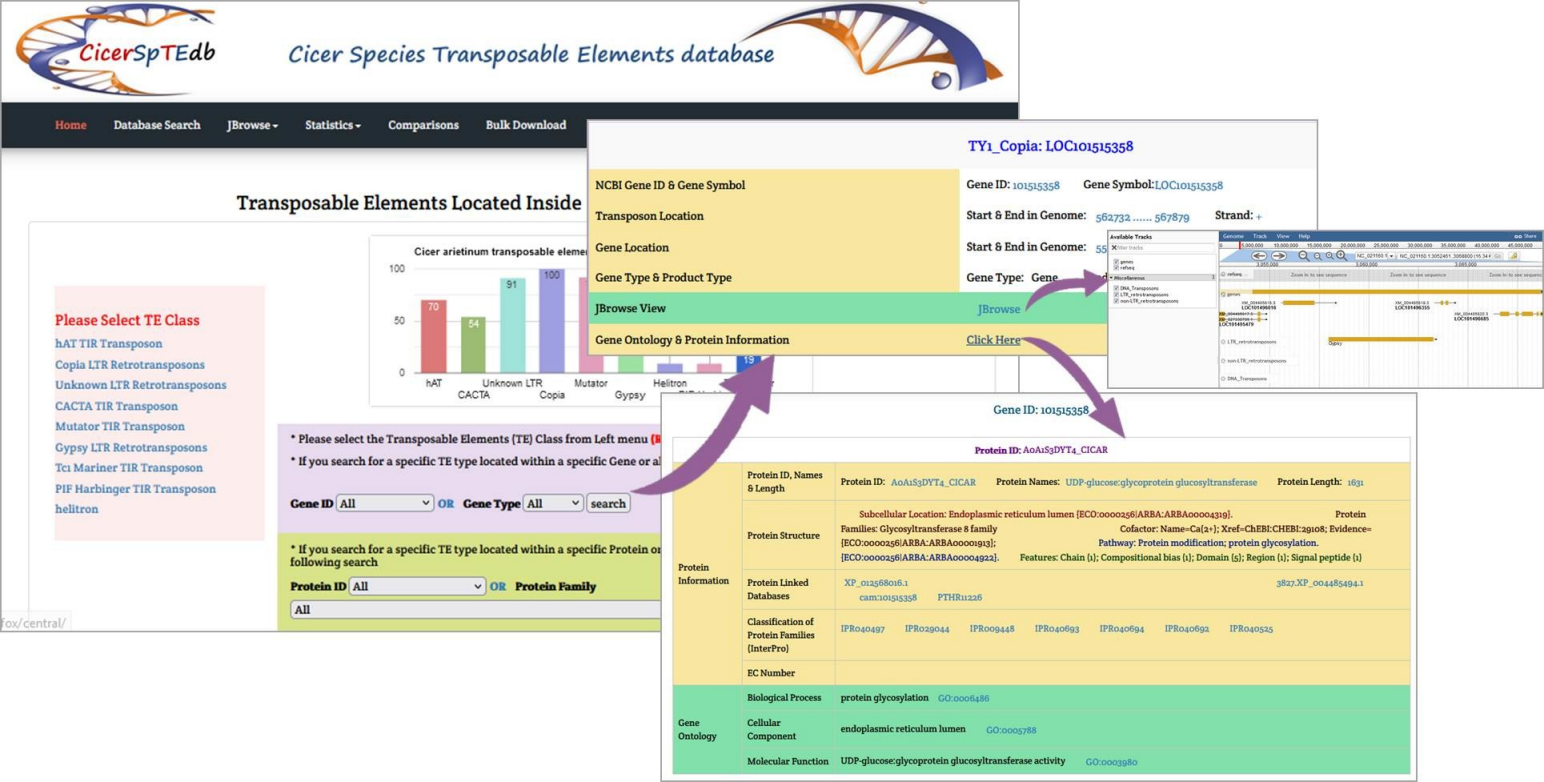
CicerSpTEdb’s search page for TEs located within genes.

On the page dedicated to TEs located within genes, the left section is used to select TE types, and the top part of the page allows users to see a statistical chart of all identified TEs located inside genes. The main section is divided into four sub-sections that allow searching by different keys. Users can search using the gene ID and gene type (gene or pseudo-gene) or using either the protein ID, protein family, or enzyme EC number. The results are displayed on a separate page and include gene details (gene ID, symbol, location, type, and product), JBrowse link, and link to gene ontology and protein information. The link will redirect the user to a new page that contains all accessible protein information such as protein ID, names, length, structure, family, EC number, externally linked databases, and gene ontology. All external databases are cross-linked, and the results can be exported by clicking the download button (Fig 8).

### The JBrowse page

**Fig 9:**
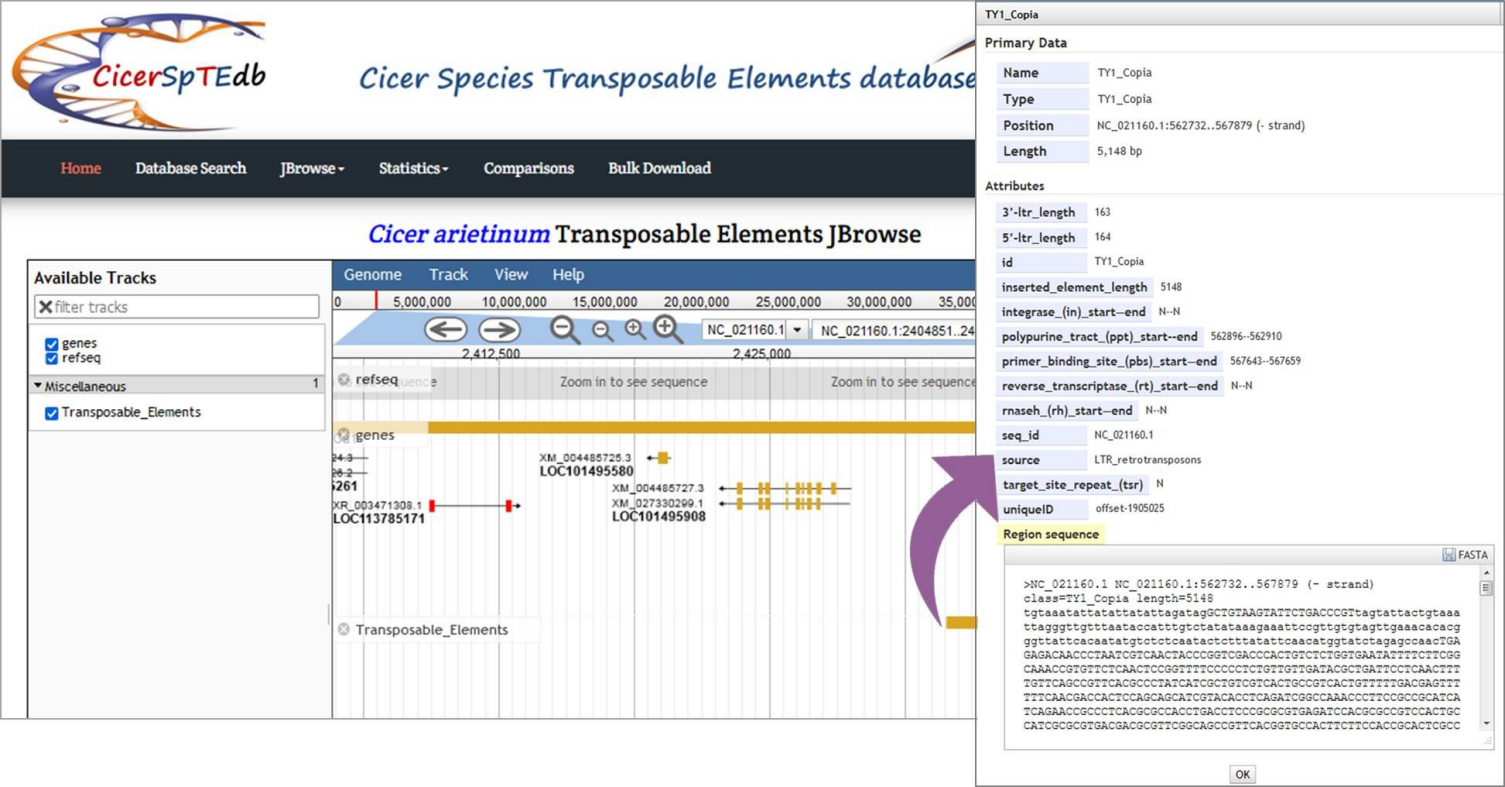
CicerSpTEdb’s JBrowse page.

JBrowse is a powerfully interactive genome visualization tool established to illustrate the coordinates of TEs in the genome. By clicking the JBrowse from the top bar of any page, a visualization window will display all chickpea coordinates, including reference sequence, genes, and identified transposons. Users can retrieve any TE’s data (name, position in the genome, length, described information, and sequence) by clicking on it in the JBrowse graphic interface. In addition, the JBrowse page offers an important function that allows users to browse all genes around TEs and the genes that TEs are positioned inside. The latter visualization function is an easy way to build a clear idea of each TE and understand the interaction between TEs and the surrounding genes (Fig 9).

### Other pages

The Statistics page was created to provide researchers with a visualization of several statistics computed from the data in CicerSpTEdb. Users can access the Statistics page through a drop-down menu that links to three pages, one for each studied genome (S7 Fig). The comparisons page was created to provide researchers with a visual comparison of identified TEs between *C*. *arietinum*, *C*. *reticulatum*, and *C*. *echinospermum*. Users can access the comparison page through the top bar from any page (S8 Fig). The Bulk Download page was created to allow researchers to download all stored data in CicerSpTEdb. The Bulk Download page allows users to select the species, transposons, and data type (fasta or gff3 files) from organism name, TE type, and data type drop-down menus (S9 Fig).

## Conclusion

CicerSpTEdb is the first comprehensive database designated to *Cicer* species transposable elements. This database contains 9761 TEs that combines DNA transposon and LTR retrotransposons. Moreover, the proposed database is available through an easy-to-use interface to allow researchers to search, browse, and download the identified TEs in *C*. *echinospermum*, *C*. *reticulatum*, and *C*. *arietinum*. We propose to continuously update the database and improve its applications to achieve its goals. We expect CicerSpTEdb to provide a valuable resource that can be used to improve our knowledge of the origin, organization, structural variations, and evolution of the *Cicer* species genomes and other related legume crops. CicerSpTEdb should help researchers develop TEs target markers for molecular breeding and to answer any related biological questions.

## Supporting information

S1 Fig The distribution of *C*. *echinospermum* TEs superfamilies according to their length. (TIF)

S2 Fig The distribution of *C*. *reticulatum* TEs superfamilies according to their length. (TIF)

S3 Fig Gene ontology of 315 *C*. *arietinum* TE-gene chimeras assigned to 486 molecular functions GO terms. (TIF)

S4 Fig Gene ontology of 215 *C*. *arietinum* TE-gene chimeras assigned to 325 cellular components GO terms. (TIF)

S5 Fig Protein-protein interaction for genes nearby TEs. (TIF)

S6 Fig Protein-protein interaction for both TE-gene chimeras and genes nearby TEs. (TIF)

S7 Fig CicerSpTEdb’s statistics page. (TIF)

S8 Fig CicerSpTEdb’s comparisons page. (TIF)

S9 Fig CicerSpTEdb’s bulk download page. (TIF)

S1 Table The details of the identified TEs in *C*. *arietinum*, including the chromosome/scaffold id, TE start and end position in the genome, TE corresponding superfamily, and TE length. (XLSX)

S2 Table The details of the identified TEs in *C*. *reticulatum*, including the chromosome/scaffold id, TE start and end position in the genome, TE corresponding superfamily, and TE length. (XLSX)

S3 Table The details of the identified TEs in *C*. *echinospermum*, including the chromosome/scaffold id, TE start and end position in the genome, TE corresponding superfamily, and TE length. (XLSX)

S4 Table The details of the identified TEs that located near genes in *C*. *arietinum*. (XLSX)

S1 File The programs and their parameters used for identification of TEs in *Cicer* species. (DOCX)
