# Supplementary figures and images for: CicerSpTEdb: A web-based database for high-resolution genome-wide identification of transposable elements in Cicer species
Source: PLoS One. 2021 Nov 11;16(11):e0259540. doi: 10.1371/journal.pone.0259540 (PMC8584679; doi:10.1371/journal.pone.0259540)

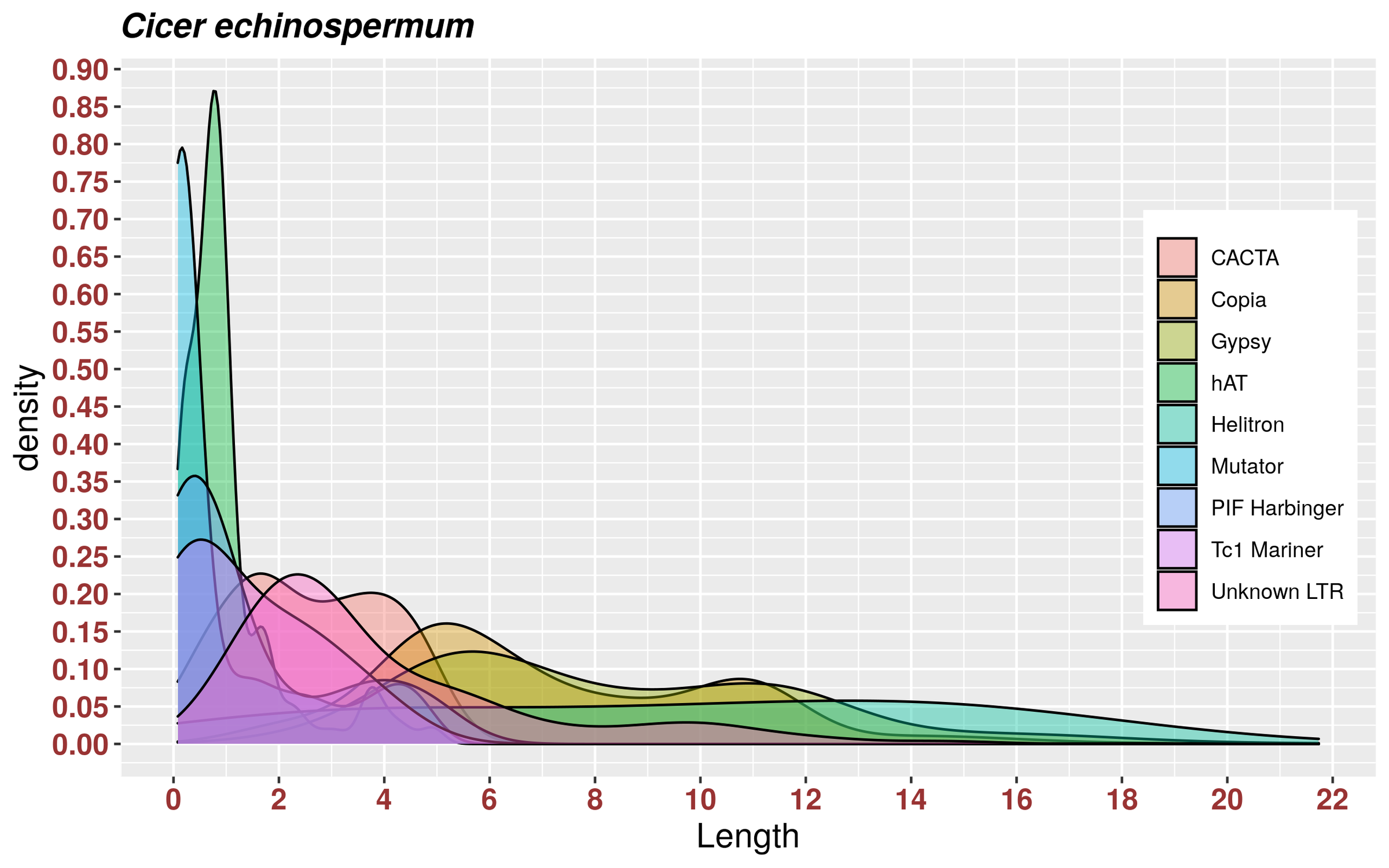

Supplement: S1 Fig — (TIF) [file pone.0259540.s001.tif]

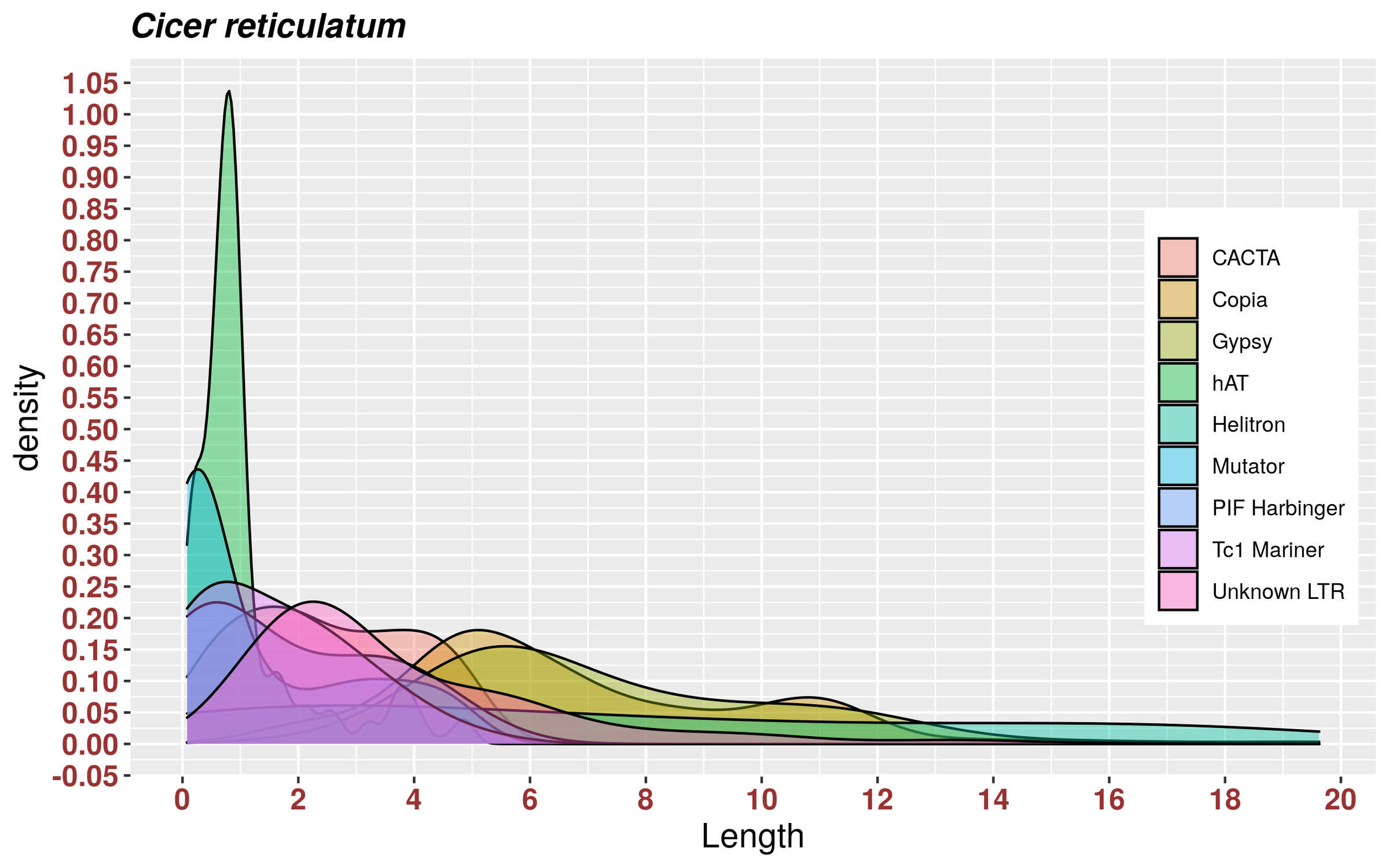

Supplement: S2 Fig — (TIF) [file pone.0259540.s002.tif]

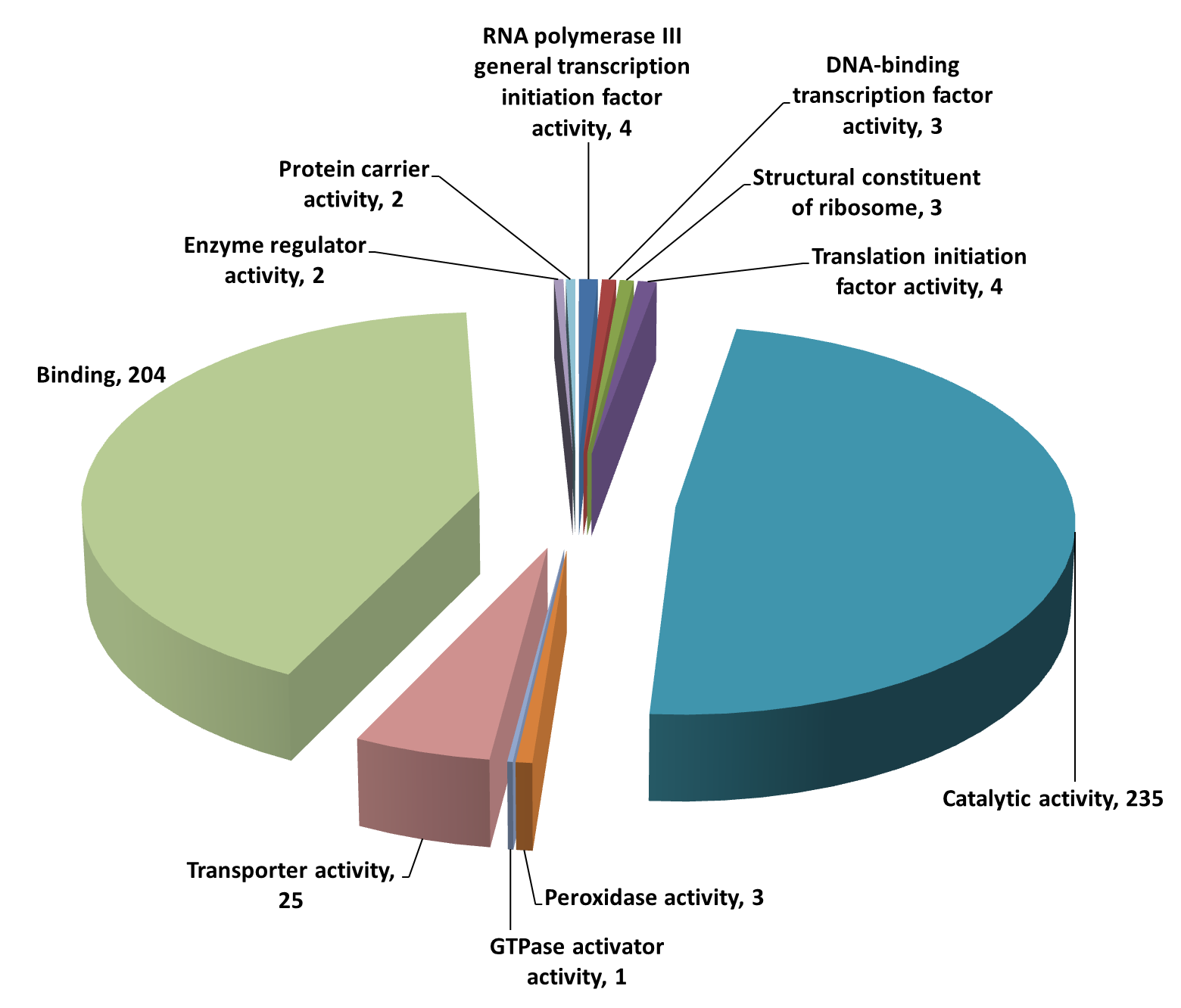

Supplement: S3 Fig — (TIF) [file pone.0259540.s003.tif]

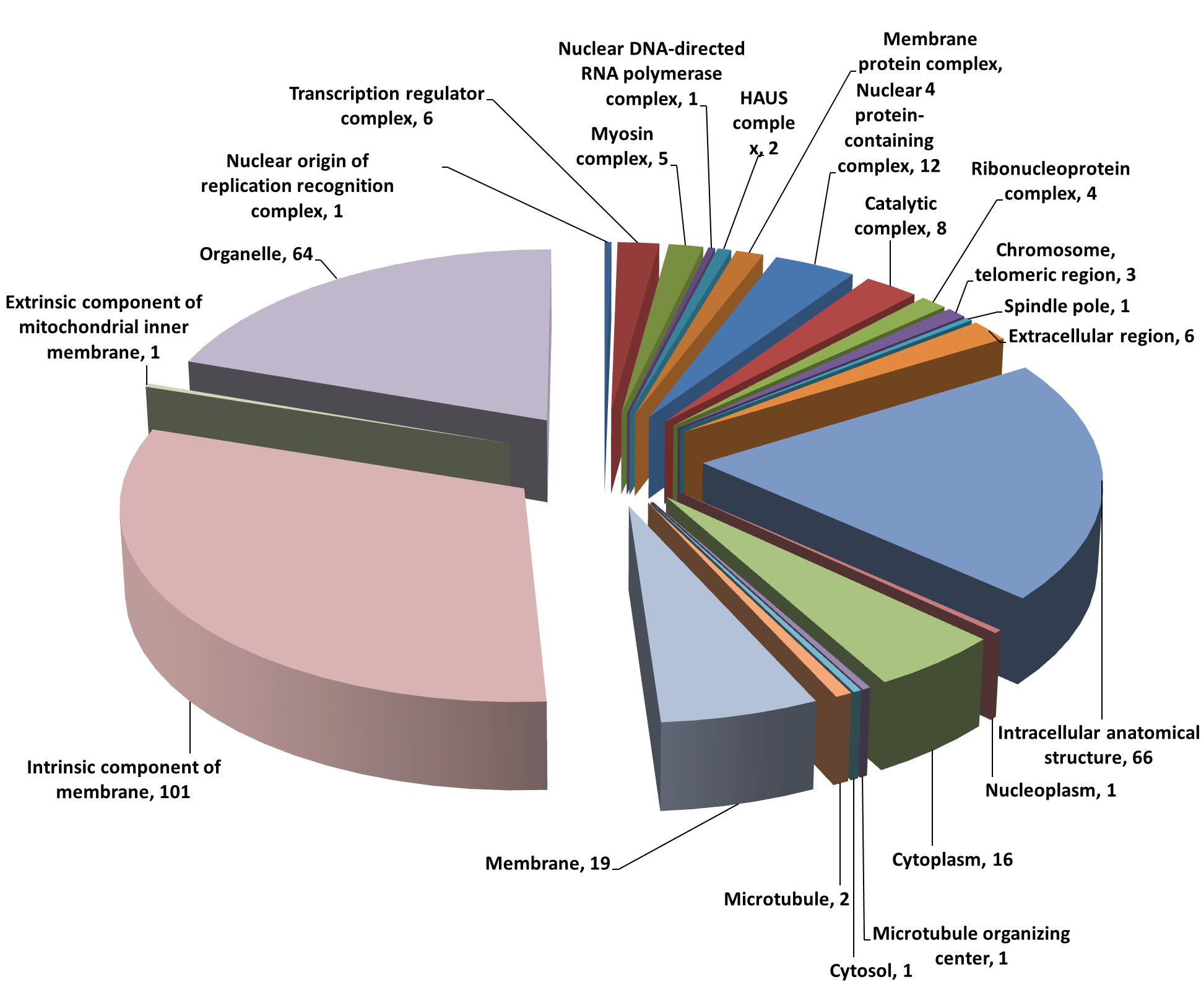

Supplement: S4 Fig — (TIF) [file pone.0259540.s004.tif]

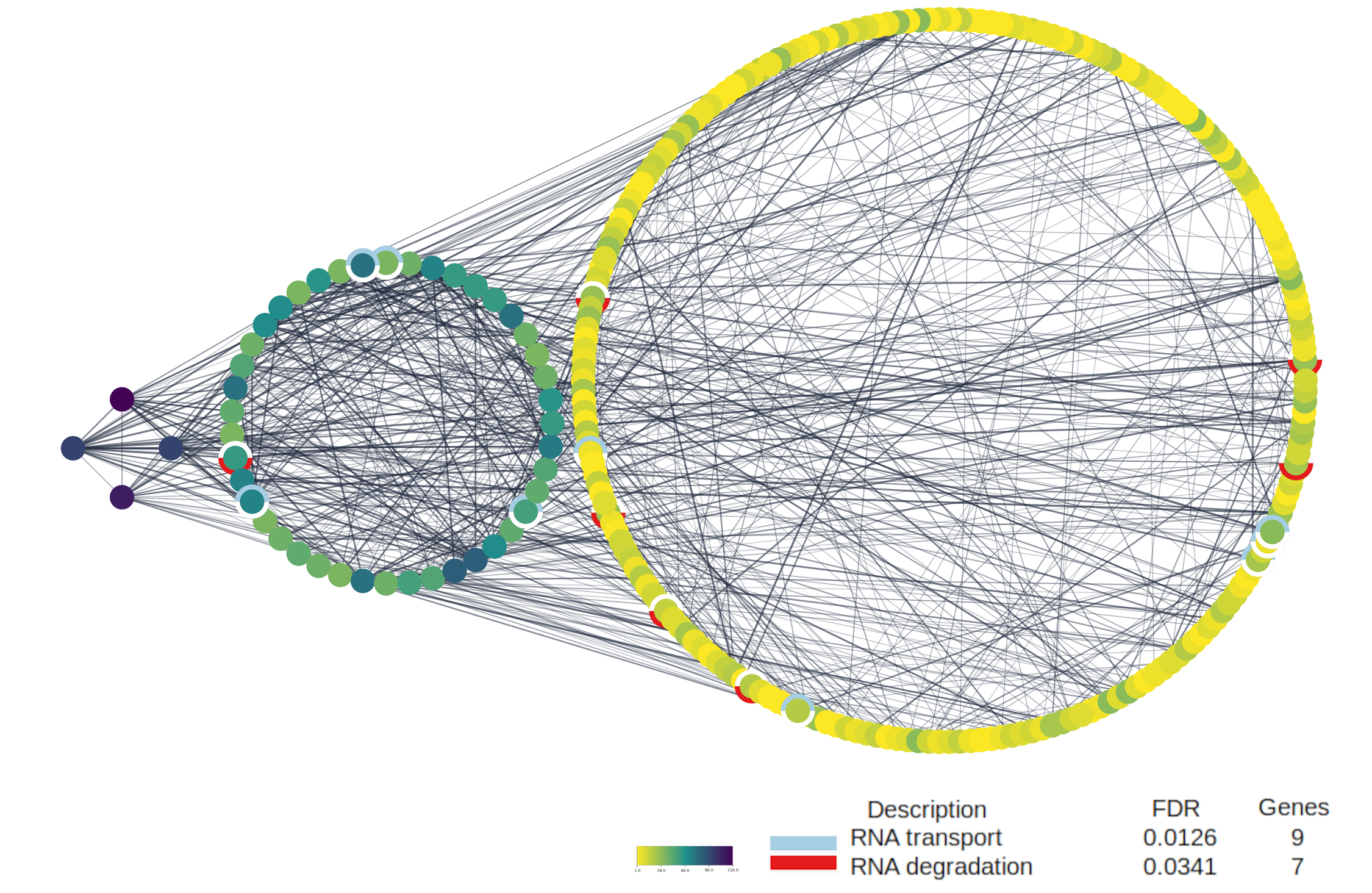

Supplement: S5 Fig — (TIF) [file pone.0259540.s005.tif]

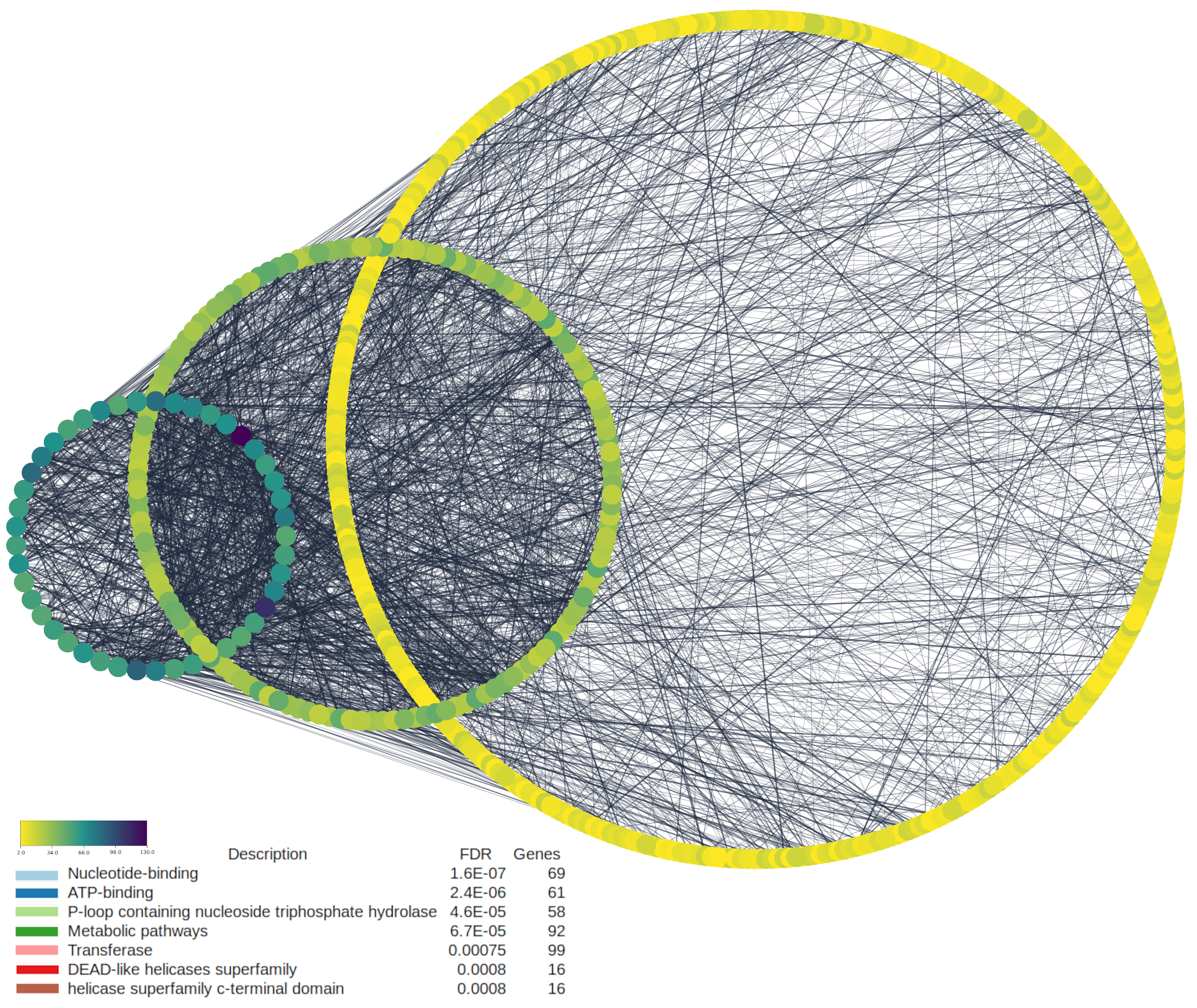

Supplement: S6 Fig — (TIF) [file pone.0259540.s006.tif]

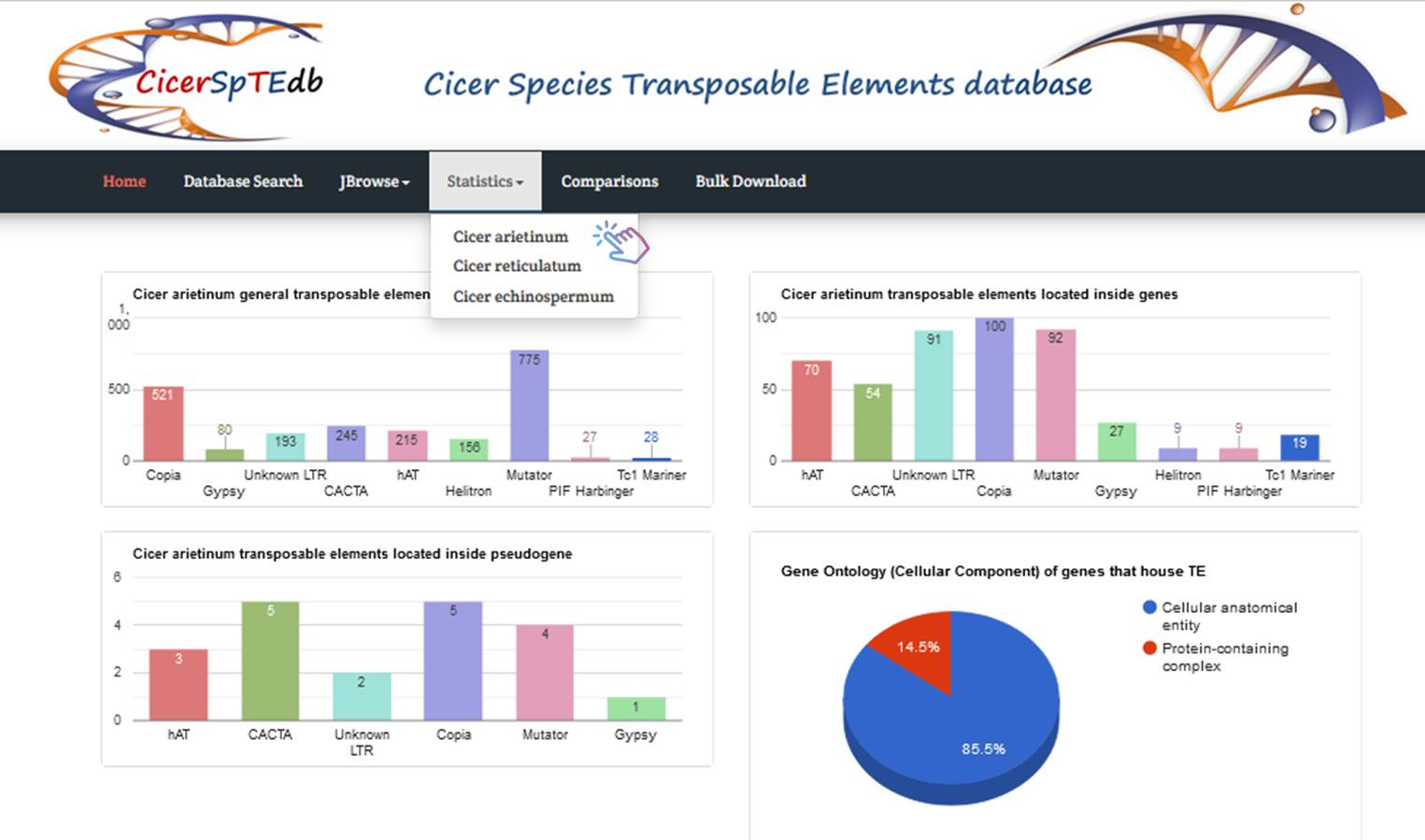

Supplement: S7 Fig — (TIF) [file pone.0259540.s007.tif]

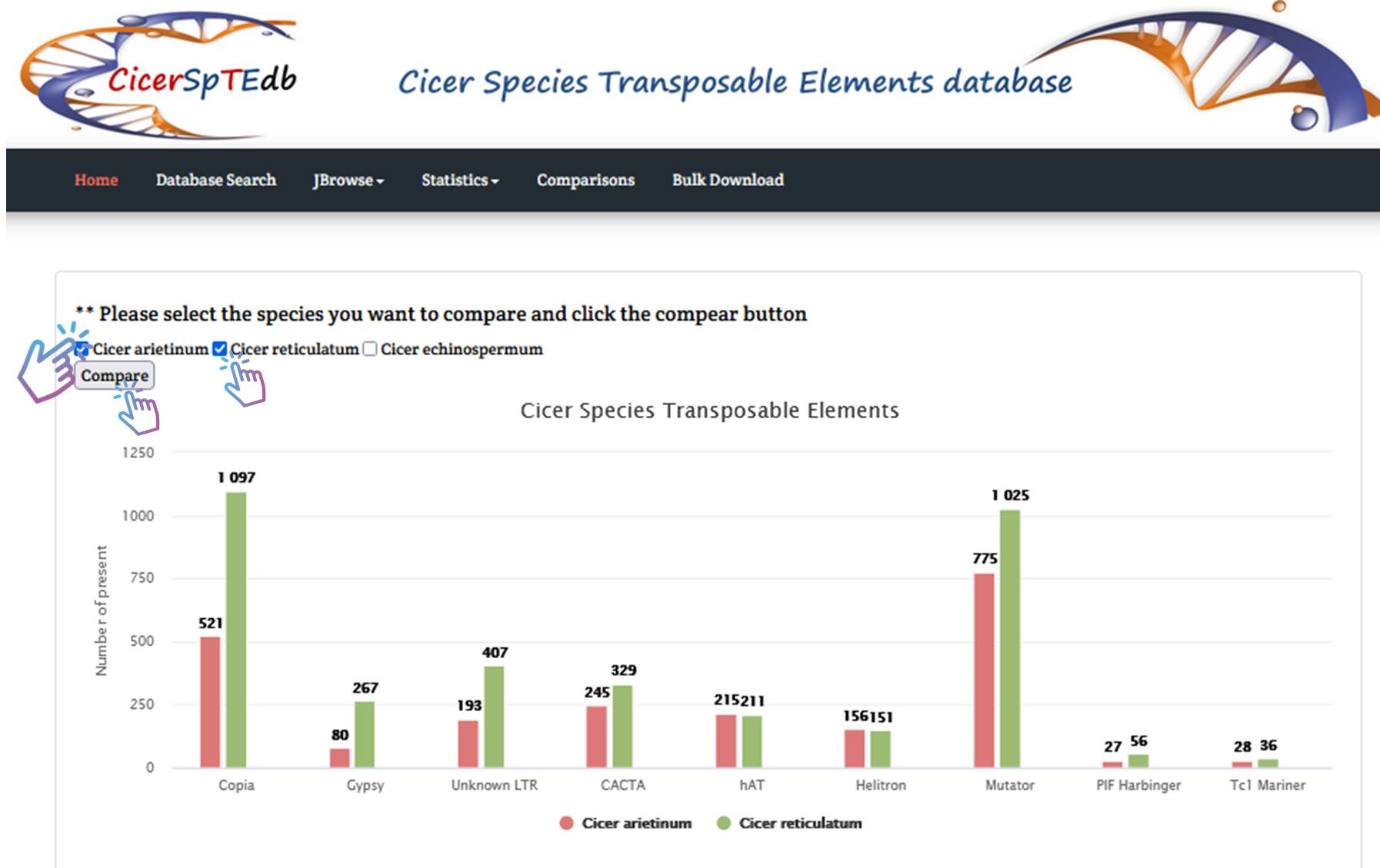

Supplement: S8 Fig — (TIF) [file pone.0259540.s008.tif]

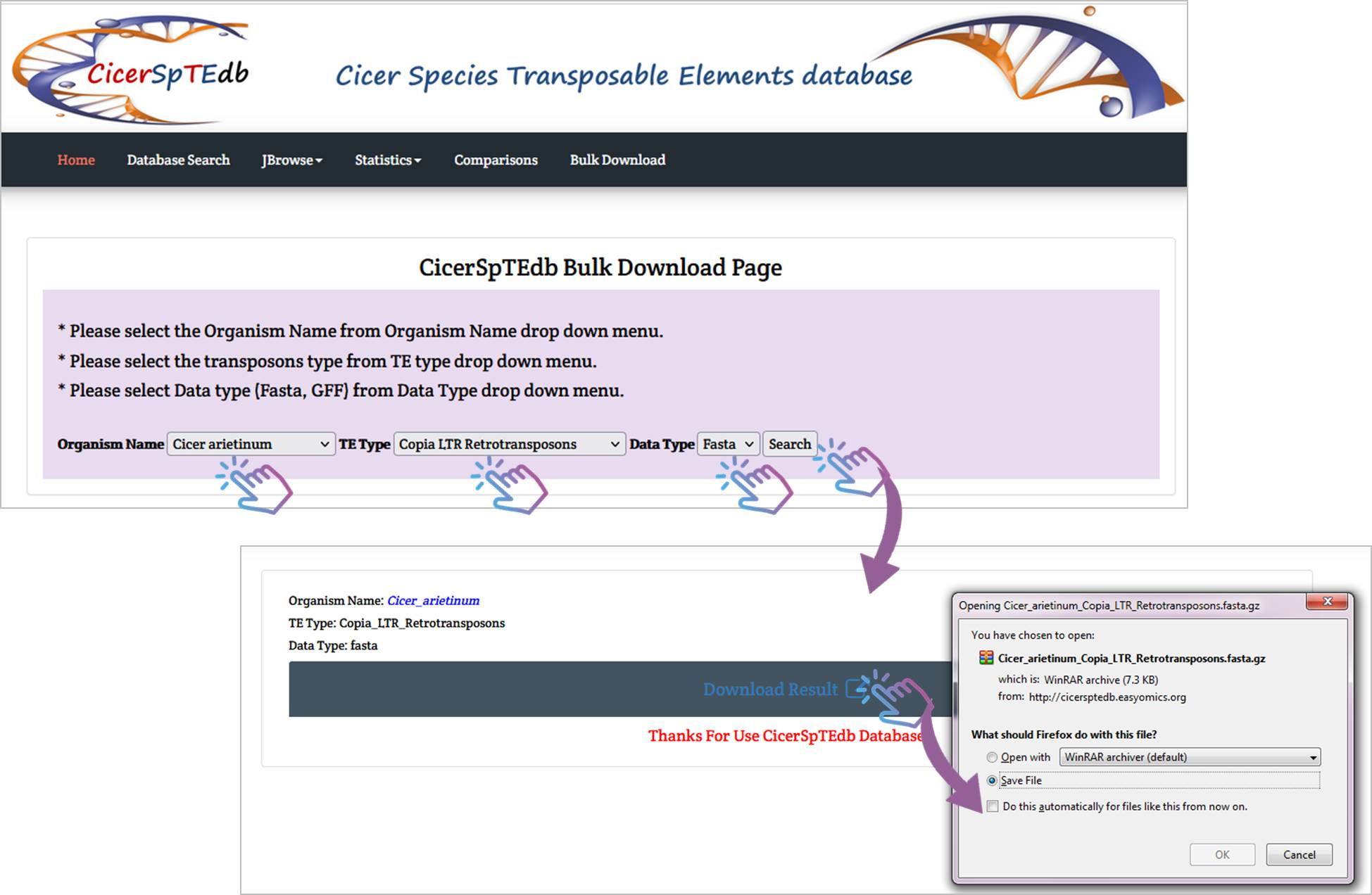

Supplement: S9 Fig — (TIF) [file pone.0259540.s009.tif]
