# Supplementary material for: CicerSpTEdb: A web-based database for high-resolution genome-wide identification of transposable elements in Cicer species
Source: PLoS One. 2021 Nov 11;16(11):e0259540. doi: 10.1371/journal.pone.0259540 (PMC8584679; doi:10.1371/journal.pone.0259540)
Supplement: S1 File — (DOCX) [file pone.0259540.s014.docx]

The EDTA pipeline combines LTRharvest[1], LTR_FINDER[2], LTR_retriever[3], Generic Repeat Finder[4], TIR-Learner[5], HelitronScanner[6], and RepeatModeler[7]. LTR_FINDER[2] version 1.0.6 was used with the minimum length of LTR regions, the maximum length of LTR regions, the maximum length of the whole candidate, and the maximum divergence between terminal repeats set to 100 bp, 7000 bp, 15,000 bp, and 85%, respectively (-w 2 -C -D 15000 -d 1000 -L 7000 -l 100 -p 20 -M 0.85) , and LTR_retriever[3] was further used to categorize the detected LTR-RTs into the subgroups of Copia-like and Gypsy-like LTR-RTs.

LTRharvest[1] version 1.5.10 was used with parameters similar to LTR_FINDER (-minlenltr 100 -maxlenltr 7000 -mintsd 4 -maxtsd 6 -similar 85 -vic 10 -seed 20 -seqids yes). Generic Repeat Finder[4] was used with parameters (-c 2 --min_tr 100 --min_space 1090 --max_space 23490 --match 2 --mismatch 2 --indel 3 -f 1 -p 30) to run *grf-main*, and used *grf-filter* to filter out extreme TDRs (*grf-filter* 100 3500 1000 20000). TIR-Learner[5] that uses machine-learning algorithms to facilitate identification and classification of TIR elements. HelitronScanner[6] the local combinational variable (LCV) algorithm to identify sequence patterns that are associated with *Helitron* transposons. The candidates that are not inserted into AT or TT target sites were filtered using the script “format_helitronscanner_out.pl” developed in EDTA toolkit. Removal of non-*Helitron* TE contamination was accomplished with the script “cleanup_tandem.pl” in EDTA toolkit with parameters “-misschar N -nc 50000 -nr 0.9 -minlen 100 -minscore 3000 -trf 1 -cleanN 1 -cleanT 1”.

**References**

1. Ellinghaus D, Kurtz S, Willhoeft U. LTRharvest, an efficient and flexible software for de novo detection of LTR retrotransposons. BMC Bioinformatics. Springer; 2008;9: 1–14. doi:https://doi.org/10.1186/1471-2105-9-18

2. Xu Z, Wang H. LTR_FINDER: an efficient tool for the prediction of full-length LTR retrotransposons. Nucleic Acids Res. 2007;35: W265–W268. doi:https://doi.org/10.1093/nar/gkm286

3. Ou S, Jiang N. LTR_retriever: a highly accurate and sensitive program for identification of long terminal repeat retrotransposons. Plant Physiol. American Society of Plant Biologists; 2018;176: 1410–1422. doi:https://doi.org/10.1104/pp.17.01310

4. Shi J, Liang C. Generic Repeat Finder: a high-sensitivity tool for genome-wide de novo repeat detection. Plant Physiol. American Society of Plant Biologists; 2019;180: 1803–1815. doi:https://doi.org/10.1104/pp.19.00386

5. Su W, Gu X, Peterson T. TIR-learner, a new ensemble method for TIR transposable element annotation, provides evidence for abundant new transposable elements in the maize genome. Mol Plant. Elsevier; 2019;12: 447–460. doi:https://doi.org/10.1016/j.molp.2019.02.008

6. Xiong W, He L, Lai J, Dooner HK, Du C. HelitronScanner uncovers a large overlooked cache of Helitron transposons in many plant genomes. Proc Natl Acad Sci. National Acad Sciences; 2014;111: 10263–10268. doi:https://doi.org/10.1073/pnas.1410068111

7. Smit AFA, Hubley R, Green P. RepeatModeler Open-1.0 (2008--2015). In: Seattle, USA: Institute for Systems Biology. [Internet]. 2015 p. 2018. Available: http://www.repeatmasker.org/RepeatModeler/
